# Inhibition of autophagy by CRMP2-derived peptide ST2-104 (R9-CBD3) via a CaMKKβ/AMPK/mTOR pathway contributes to ischemic postconditioning-induced neuroprotection against cerebral ischemia-reperfusion injury

**DOI:** 10.1186/s13041-021-00836-0

**Published:** 2021-08-06

**Authors:** Yuan Yao, Yingshi Ji, Jinghong Ren, Huanyu Liu, Rajesh Khanna, Li Sun

**Affiliations:** 1 Department of Pharmacology, College of Basic Medical Sciences, Jilin University, Changchun, Jilin 130021 People’s Republic of China; 2 Department of Pharmacology, College of Medicine, University of Arizona, 1501 North Campbell Drive, P.O. Box 245050, Tucson, AZ 85724 USA; 3 Department of Neurology and Neuroscience Center, The First Hospital, Jilin University, Changchun, Jilin 130021 People’s Republic of China

**Keywords:** Cerebral ischemia injury, Glutamate, CRMP2, Apoptosis, Autophagy, CaMKKβ/AMPK/mTOR pathway

## Abstract

Cerebral ischemia, a common cerebrovascular disease, is characterized by functional deficits and apoptotic cell death. Autophagy, a type of programmed cell death, plays critical roles in controlling neuronal damage and metabolic homeostasis, and has been inextricably linked to cerebral ischemia. We previously identified a short peptide aptamer from collapsin response mediator protein 2 (CRMP2), designated the Ca^2+^ channel-binding domain 3 (CBD3) peptide, that conferred protection against excitotoxicity and traumatic brain injury. ST2-104, a nona-arginine (R9)-fused CBD3 peptide, exerted beneficial effects on neuropathic pain and was neuroprotective in a model of Alzheimer’s disease; however, the effect of ST2-104 on cerebral ischemia and its mechanism of action have not been studied. In this study, we modeled cerebral ischemia–reperfusion injury in rats with the middle cerebral artery occlusion (MCAO) as well as challenged SH-SY5Y neuroblastoma cells with glutamate to induce toxicity to interrogate the effects of ST2-104 on autophagy following ischemic/excitotoxic insults. ST2-104 reduced the infarct volume and improved the neurological score of rats subjected to MCAO. ST2-104 protected SH-SY5Y cells from death following glutamate exposure via blunting apoptosis and autophagy as well as limiting excessive calcium entry. 3-Methyladenine (3-MA), an inhibitor of autophagy, promoted the effects of ST2-104 in inhibiting apoptosis triggered by glutamate while rapamycin, an activator of autophagy, failed to do so. ST2-104 peptide reversed glutamate-induced apoptosis via inhibiting Ca^2+^/CaM-dependent protein kinase kinase β (CaMKKβ)-mediated autophagy, which was partly enhanced by STO-609 (an inhibitor of CaMKKβ). ST2-104 attenuated neuronal apoptosis by inhibiting autophagy through CaMKKβ/AMPK/mTOR pathway. Our results suggest that the neuroprotective effect of ST2-104 are due to actions on the crosstalk between apoptosis and autophagy via the CaMKKβ/AMPK/mTOR signaling pathway. The findings present novel insights into the potential neuroprotection of ST2-104 in cerebral ischemia.

## Introduction

Cerebral ischemia is a cerebrovascular disorder and the second leading cause of death globally, killing approximately 5.5 million people annually [1]. It is characterized by key molecular events including excitotoxicity, calcium overload, and overproduction of free radicals—all of which culminate in neuronal and glial apoptosis [2]. Although thrombolysis is reportedly an effective treatment for cerebral ischemia, other neuroprotective strategies merely reduce the intensity of symptoms [3, 4]. The recognition that brief periods of ischemia trigger complex cellular events leading to progressive apoptotic necrotic neuronal cell death has motivated intense research efforts to identify compounds/biologics to curb apoptosis to manage cerebral ischemia.

Decades of research has implicated apoptosis as a prime regulator of neuronal death following cerebral ischemia [5]; however, the potential mechanisms have not been fully elucidated. Autophagy, a specialized form of cell death, acts in concert with necrosis and necroptosis, to regulate apoptosis [6]. During autophagy, cytoplasmic proteins are sequestered into double-membrane vesicles called autophagosomes, then fuse with lysosomes to produce single-membraned autophagolysosomes, and degraded by lysosomal hydrolases; thus, autophagy contributes to both the maintenance of normal cellular metabolism and renewal of organelles [7].

Apoptosis-related genes B-cell leukemia-2 (Bcl-2), Bcl-xl, and Bcl-2-associated X protein (Bax) mediate the crosstalk between autophagy and apoptosis [8]. Caspases have been shown to directly interact with core autophagy proteins [9, 10]. Liu and colleagues reported that following long-term administration of baclofen (a GABAβ receptor agonist), neuronal injury was alleviated via inhibition of autophagy [11]. In that study, the Bcl-2/Bax ratio increased and a signaling pathway involving protein kinase B (Akt/PKB), glycogen synthase kinase 3β (GSK-3β), and extracellular signal–regulated kinase (ERK) also increased [11]. Zheng and co-workers reported that rats subjected to middle cerebral artery occlusion (MCAO) had high levels autophagy in ischemic brain regions as evidence by increased levels of Caspase-3/Beclin-1 double-labeled positive cells. The formation of autophagolysosomes, which contain beclin-1, was also enhanced following MCAO, ultimately leading to cellular demise [12]. Together, these findings suggest that inhibition of apoptosis may attenuate cerebral ischemia by regulating autophagy [7, 13].

It has been reported that Ca^2+^ overload caused by pathological levels of the excitatory neurotransmitter glutamate following ischemic events results in neuronal cell death, a phenomenon referred to as excitotoxicity [14, 15]. The serine/threonine-specific calcium/calmodulin-dependent protein kinase kinase (CaMKK), α and β isoforms, are activated by high intracellular calcium. As a result, downstream targets of CaMKK including Calcium/calmodulin-dependent protein kinase I, isoform I (CaMKI), CaMKIV and AMP-activated protein kinase (AMPK) can be phosphorylated by activated CaMKK [16]. The activated AMPK directly phosphorylates Ser317 or Ser777 of Unc-51 Like Autophagy Activating Kinase 1 (ULK1) to initiate autophagic processes [17]. Mammalian target of rapamycin (mTOR) maintains nutrient utilization via sensing ATP and amino acid levels in the growth of cells. AMPK-ULK1 complex can be suppressed by increased mTOR activity which may further blunt autophagy [18]. Sun and co-workers found that in neurons challenged with oxygen glucose deprivation/reperfusion (OGD/R), propofol regulates a Ca^2+^/CaMKKβ/AMPK/mTOR signaling platform that contributes to regulation and inhibition of autophagy [19, 20].

Previous work by our groups have identified a Ca^2+^ channel-binding domain 3 (CBD3) peptide (sequence ARSRLAELRGVPRGL) from the cytosolic collapsin response mediator protein 2 (CRMP2) with anti-apoptotic/neuroprotective properties in a cellular model of Alzheimer's disease, properties ascribed to the propensity of CBD3 to attenuate excessive influx of Ca^2+^ and to inhibit apoptosis [21]. A nona-arginine (R9)-fused cell-penetrant variant of CBD3 peptide, designated here as ST2-104 [22], blocks calcium influx by interfering with interactions of CRMP2 with N-methyl-D-aspartate receptors, N-type voltage-gated calcium channels (CaV2.2), and sodium/calcium exchangers [22–27]. Whether ST2-104 regulates apoptosis and autophagy following cerebral ischemia injury is unknown. In this study we interrogated if ST2-104 could inhibit neuronal apoptosis by inhibiting autophagy and mapped the signaling pathways involved using an in vivo model of cerebral ischemia–reperfusion injury (MCAO) as well as in vitro model of glutamate-triggered excitotoxicity (SH-SY5Y neuroblastoma cells).

## Materials and methods

### Animals

Adult male Sprague–Dawley rats were purchased from Changchun Yisi Experimental Technology Co. Ltd. Animals weighing 250–300 g were housed in individual cages at 25 °C and had access to free water and food. All procedures were performed in strict adherence to the Ethics Committee of Jilin University.

### Peptides

A nona-arginine (R9)-coupled variant of CBD3 peptide (ARSRLAELRGVPRGL) was synthesized and HPLC purified by GenScript Inc (China). Peptides were dissolved in deionized water or DMSO (Sigma-Aldrich, St Louis, MO), aliquoted, and stored at − 70 °C. All chemicals, unless noted, were purchased from Sigma-Aldrich. Scramble and random sequence‐based peptides conjugated to various cargoes as controls have been previously studied as controls in molecular, biochemical and behavioral assays and demonstrated to have no effects [28–31].

### Focal cerebral ischemia-middle cerebral artery occlusion (MCAO) model

We used the middle cerebral artery occlusion (MCAO) method to induce 120 min of ischemia followed by 24 reperfusion [32]. Briefly, rats were anesthetized with chloral hydrate (0.4 ml/100 g, intraperitoneal injection). We then separated the external carotid artery (ECA) and internal carotid artery (ICA) along the common carotid artery (CCA), ligated the proximal end of the CCA and ECA, and prepared a thread at the distal end of the CCA. Then, we clamped the ICA with an arterial clip temporarily, made a small cut at 4 mm from the bifurcation of the CCA, inserted a nylon filament suture (Beijing Cinontech Co. Ltd. Beijing, China, 0.36 ± 0.02 mm) gently until it entered into the ICA, and then fixed the nylon filament with a thread around the distal end of the CCA. Rats were housed singly following the MCAO surgery.

### In vivo treatments

Twenty-four (six per group) rats were randomly assigned to one of 4 groups: (i) Sham group (rats received the same surgical exposure procedures as rats in the MCAO group without occlusion of ICA); (ii) MCAO group; (iii) MCAO + 3 mg/kg ST2-104 peptide (Low or L-STS-204); or (iv) MCAO + 15 mg/kg ST2-104 peptide (High or H-ST2-204). Sham and MCAO rats were administered equal amount of normal saline intraperitoneally as control. Rats in the drug intervention groups were intravenously administered ST2-104 peptide (Qiangyao biological company, Shanghai, China) daily for 7 consecutive days before MCAO).

### Neurological score

Twenty-four hours following the sham/MCAO surgeries, rats were subjected to a neurological examination (Longa test) consisting of a six-point scale [33]: 0, no symptom; 1, Failure to extend left forepaw completely (shows mild focal neurological deficit); 2, circling/turning to one side while walking (moderate focal neurological deficit); 3, falling to one side while walking (indicates a severe focal neurological deficit); 4, loss of consciousness and no voluntary movement; and 5, death due to brain ischemia.

### Triphenyl tetrazolium chloride (TTC) staining and quantification of infarct volume

Twenty-four hours following the sham/MCAO surgeries, rats (n = 6 for each group) were anesthetized and decapitated. Their brains were sliced into five or six 2-mm coronal sections and submerged in freshly prepared TTC (Sigma-Aldrich, St. Louis, USA) solution for 30 min at 37 °C without light, followed by immersed in 4% formalin for 4 h. Images of brain slices were taken and recorded after staining. Image J software was applied to measure the infarct areas and the whole area of each coronal section. The infarct volume was calculated as follow: Infarct volume (%) = (V_1_ + V_2_ + … + V_5_)/(M_1_ + M_2_ + … + M_5_) × 100%, where V_n_ represents the infarct volume of each slice, M_n_ represents the total volume of each slice.

### Glutamate-induced excitotoxicity and MTT cell viability assay

SH-SY5Y neuroblastoma cells, a cell line derived from the SK-N-SH neuroblastoma cells (Key Gen Biotech Co. Ltd. Jiangsu, China), were grown in Dulbecco’s modified Eagle’s medium (DMEM) (Gibco, Shanghai, China) supplemented with 10% fetal bovine serum (FBS) (Biological Industries, 04–001-1ACS, Israel) at a constant temperature at 37 °C with 5% CO2. The cells were plated in 96-well plates (density: 5 × 103/well) until cell confluence reached ~ 60%. Cells were then challenged with different concentrations of glutamate (1, 2, 4, 8, 10, 20, 40, 80 mM) and 100 μM D-serine (a NMDAR co-agonist) for 24 and 48 h; ST2-104 (0.1, 1, 5, 10, 25, 50, 100 μM) for 24 h and 48 h; or Glu (20 mM) and 100 μM D-serine with ST2-104 peptide (3, 10, 30 μΜ; pre-treatment 30 min) for 24 h.

Following these treatment conditions, the culture medium was discarded and 20 µl 3-[4,5-dimethylthiazole-2-yl]-2,5-diphenyltetrazolium bromide (MTT) (Sigma-Aldrich, St. Louis, USA) solution was added to each well at 37 °C for 4 h. DMSO (150 μl/well) was added for 10 min to dissolve the purple crystals; MTT is a yellow tetrazolium dye that turns purple when it is reduced to an insoluble formazan with DMSO. Finally, the optical density values were examined using a microplate reader (Tecan, Switzerland) at 490 nm.

### Cell culture and drug administration

In some experiments, SH-SY5Y cells were treated with 1 mM 3-Methyladenine (3-MA), an inhibitor of autophagy, (Sigma-Aldrich, St. Louis, USA); 500 nM rapamycin (Selleck Chemicals, Houston, TX); or 10 μM STO-609, an inhibitor of CaMKKβ (Selleck Chemicals, Houston, TX) for 24 h with or without ST2-104.

### Hoechst 33258 staining

To detect apoptotic cells, the morphology of nuclear chromatin was assessed using the Hoechst 33258 kit (Beyotime, Shanghai, China). Following treatment as indicated above, cells in 6-well plates were washed with PBS three times and stained with 200 μM Hoechst 33258 for 10 min. Subsequently, apoptotic cells were detected with fluorescence microscopy (Olympus, Japan). The morphology of the apoptotic cell nucleus appears compact with dense staining. The dye stains condense chromatin of apoptotic cells more brightly than chromatin of normal cells.

### Monodansylcadaverine (MDC) staining for autophagosome formation

Autophagic morphological changes were evaluated by fluorescence microscopy using monodansylcadaverine (MDC) staining. MDC is a specific marker for autophagosome formation [34]. After the indicated treatments, cells were rinsed with PBS for 3 times and incubated in 200 µl of 0.05 mM MDC (Solarbio, Beijing, China) at 37 °C for 30 min. Then, the cells were rinsed with PBS for 2 times and the aggregation of autophagic vacuoles was observed under a fluorescence microscope with an excitation wavelength of 460–500 nm and an emission wavelength of 512–542 nm.

### Intracellular Ca^2+^ measurements

Intracellular Ca^2+^ concentration was monitored by the fluorescent dye Fluo-3-acetoxymethyl ester probe (Fluo-3AM) (Beyotime, Shanghai, China). After treatment, cells in 6-well plates were washed with PBS three times and incubated with 5 μM Fluo-3AM for 1 h at 37 °C. Next, the cells were washed 3 times with PBS. Upon excitation at 488 nm, the fluorescence was monitored at 525 nm wavelength using a flow cytometer (Bio-Rad). The mean fluorescent intensity (MFI) represents reflects intracellular Ca^2+^ concentration.

### Western blot analysis

After drug treatment, we used radioimmunoprecipitation RIPA (Beyotime, Shanghai, China) buffer to collect and lyse the cerebral tissues or cells on ice for 30 min. After centrifugation, protein samples were analyzed by BCA kit (Solarbio, Beijing, China). Samples (30 μg protein) were resolved in 10%-15% SDS-PAGE with electrophoresis. PVDF membranes (Bio-Rad) were used to transfer. 5% non-fat milk was used to block the membranes for 2 h and then proteins on membranes were probed with following primary antibodies: β-actin (1:5000, Sigma-Aldrich, St. Louis, USA), Bax, Bcl-2, caspase-3, Beclin-1, mTOR, p-AMPK, AMPK (1:1000, Cell Signaling Technology, Inc., Danvers, MA, USA), CaMKKβ, LC3-II, p-mTOR (1:1000, Abcam Cambridge, UK) overnight at 4 °C. Next, proteins on membranes were reacted with the appropriate secondary antibodies (anti-rabbit or anti-mouse IgG, 1:1000; Beyotime) for 2 h at 25 °C. Between antibody incubations, membranes were washed in TBST three times. The protein bands were detected by the ECL reagent (Bio-Rad) and gray values were analyzed by image J software.

### Statistics

One-way analysis of variance (ANOVA), followed by Tukey post-hoc test, was used to analyze significant differences. All data shown in the results were expressed as the mean ± SEM. Data were carried out with GraphPad Prism 8.0 (GraphPad Software, USA). The statistical significance was defined as *P* < 0.05.

## Results

### R9-CBD3, a CRMP2-derived peptide (i.e., ST2-104), decreases brain infarction and enhances neurological function in MCAO rats

**Fig. 1:**
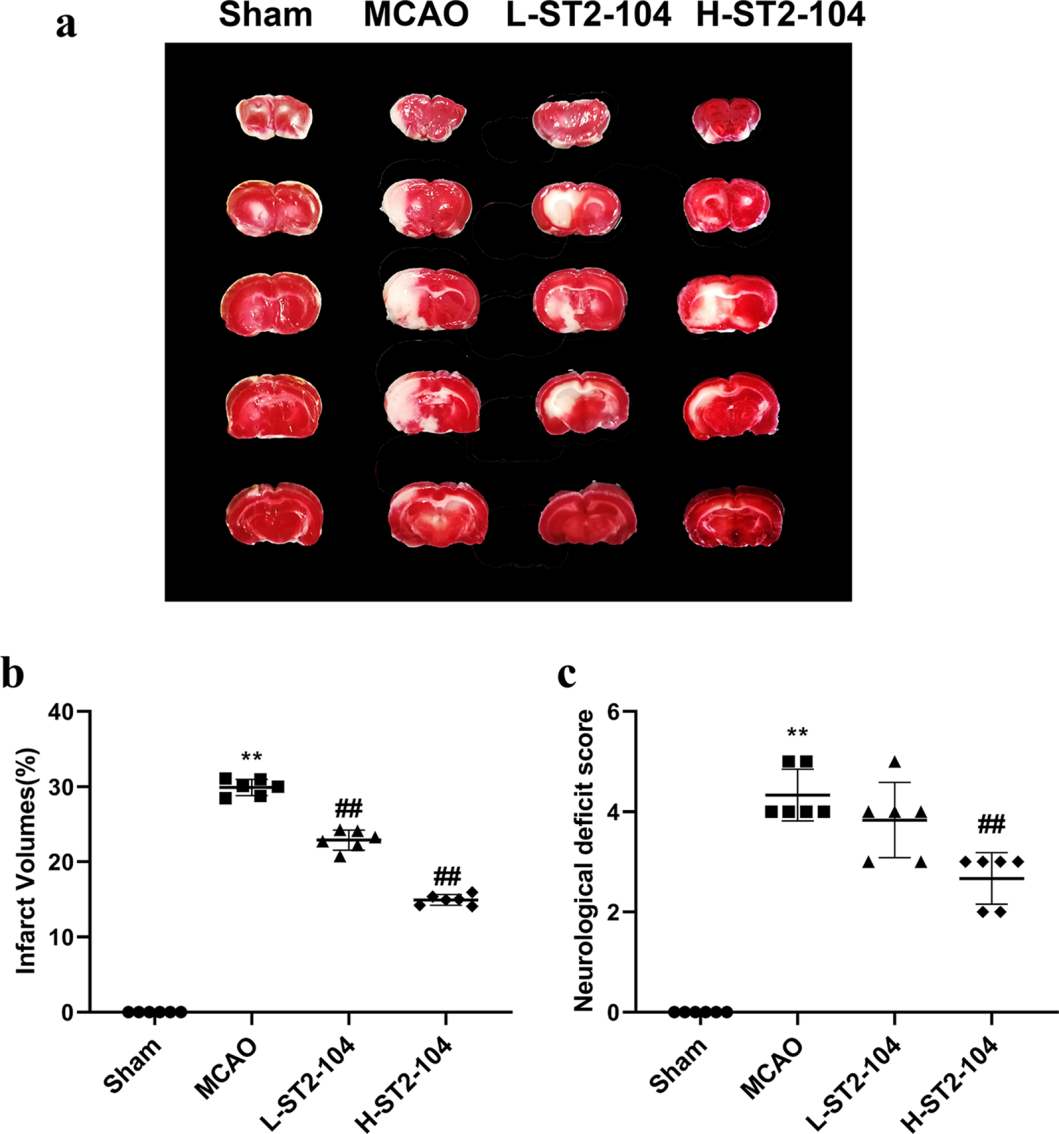
ST2-104 decreases brain infarction and enhances neurological function in MCAO rats. **a** Representative coronal sections stained with triphenyl tetrazolium chloride (TTC) from rat brains in the sham, MCAO, MCAO + 3 mg/kg ST2-104 (L-ST2-104), and MCAO + 15 mg/kg ST2-104 (H-ST2-104) groups. TTC-stained red color indicates normal region, and white color is an infarct lesion (*n* = 6/group). **b** Bar graphs (with individual data points as indicated) of ischemic infarct volume measured following 24 h of the four groups as indicated (*n* = 6/group). **c** Quantification of the six-point scale neurological score of the four groups as indicated (*n* = 6/group). Scoring was performed by investigators blinded to the experimental condition. Data are presented as Mean ± SEM, analyzed by one-way analysis of variance (ANOVA). ^**^P < 0.01 vs. Sham group; ^##^*P* < 0.01 vs. MCAO group

Twenty-four hours following MCAO, we evaluated the extent of cerebral damage using triphenyl tetrazolium chloride (TTC), which distinguishes between ischemic (white) and non-ischemic (red) areas (Fig. 1a). Considerable infarct changes were observed in the MCAO group (29.1 ± 2) as compared to sham-operated group (p < 0.01). Both the low ST2-104 and high ST2-104 dose-treated groups showed a significant reduction in infarct volume (Fig. 1b). Sensorimotor functions were assessed in these groups revealing a severe neurological defect in the MCAO group relative to sham control (p < 0.01), as shown by a lesser composite score (Fig. 1c). This neurological score was significantly improved by treatment with ST2-104 (high dose) group (p < 0.01) compared to the MCAO group (Fig. 1c). These results indicate that pretreatment with ST2-104 peptide could modulate infarct size and attenuate the neurological deficits inflicted by cerebral ischemia‑reperfusion injury.

### ST2-104 peptide inhibits apoptosis and autophagy in ischemic brain tissues of MCAO rats

**Fig. 2:**
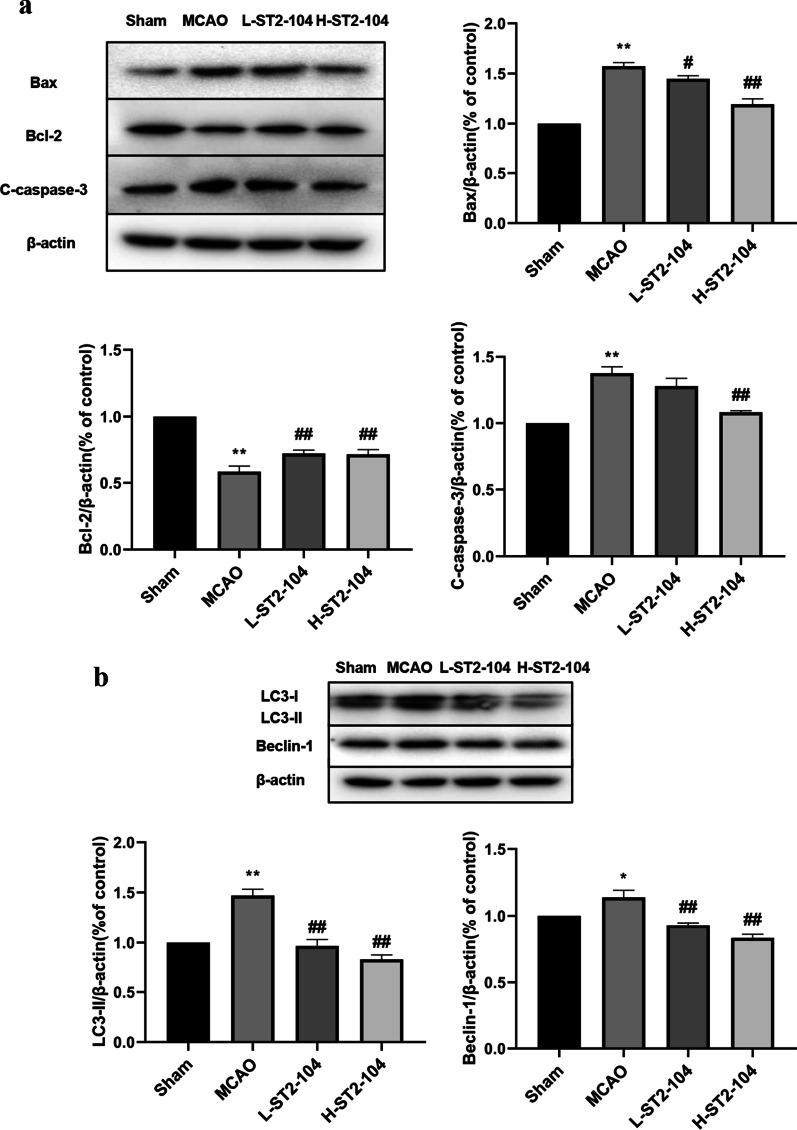
ST2-104 inhibits apoptosis and autophagy related proteins in ischemic brain tissues of MCAO rats. Rats were subjected to 120 min ischemia followed by 24 h reperfusion. Extracts from the sham-operated and ischemic cerebral cortex were separated for immunoblotting. **a** Changes of Bax, Bcl-2 and C-caspase-3 and **b** LC3-I/II and Beclin 1 expressions in the different groups. Levels of β-actin protein were used as the loading control. Bar represents mean ± SEM from 3 rats in each group. ^*^P < 0.05, ^**^P < 0.01 vs. Sham group; ^#^*P* < 0.05, ^##^*P* < 0.01 vs. MCAO group with one-way ANOVA with Tukey’s post-hoc test

We measured the expression of apoptosis-related and autophagy-related proteins in ischemic cerebral tissues (i.e., 24 h following MCAO) of rats by Western blot. In comparison to the MCAO group, the levels of Bax and C-caspase-3 were decreased in the ST2-104 groups, especially in the H-ST2-104 group. Similarly, the expression of Bcl-2 was increased in the ST2-104 pretreated groups in comparison with the MCAO group (Fig. 2a).

Next, we measured the levels of LC3, another key protein in the autophagic cascade. LC3, the microtubule-associated protein 1A light chain 3, exists in cytosolic form (LC3-I) and membrane-bound form (LC3-II). The ratio of conversion from LC3-I to LC3-II is closely correlated with the extent of autophagosome formation. Alternatively, autophagosome numbers are widely assessed by quantifying LC3-II puncta numbers in cells or via immunoblotting. Compared with the control/sham group, the levels of LC3-II and Beclin-1were reduced in the ST2-104 pretreated groups, especially in the H-ST2-104 group (Fig. 2b). These results show that ST2-104 pretreatment reduces level of proteins involved in neuronal apoptosis and autophagy in rats with a cerebral ischemia‑reperfusion injury.

### ST2-104 peptide attenuates CaMKKβ/AMPK/mTOR pathway in MCAO rats

**Fig. 3:**
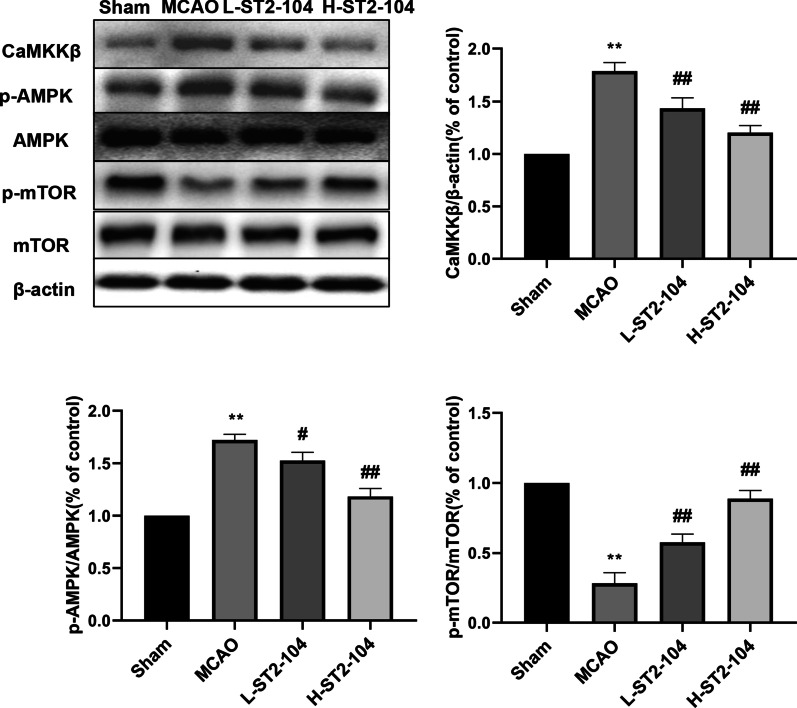
ST2-104 affects the CaMKKβ/AMPK/mTOR pathway in ischemic brain tissues of MCAO rats. Rats were subjected to 120 min ischemia followed by 24 h reperfusion. Extracts from the sham-operated and ischemic cerebral cortex were separated for immunoblotting. Detection of CaMKKβ, p-AMPK and p-mTOR protein levels using Western blot analysis. Levels of β-actin protein were used as the loading control. Bar represents mean ± SEM from 3 rats in each group. ^**^P < 0.01 vs. Sham group; ^#^*P* < 0.05, ^##^*P* < 0.01 vs. MCAO group with one-way ANOVA with Tukey’s post-hoc test

To get additional insight into the molecular pathways involving autophagy/apoptosis, we determined the levels of key autophagy related proteins. We observed an increase in the level of CaMKKβ and p-AMPK/AMPK ratio and a decrease in the p-mTOR/mTOR ratio in samples from rats with MCAO (Fig. 3). Pretreatment with ST2-104 reversed these changes (Fig. 3). Therefore, the antiapoptotic effects of ST2-104 in the face of a cerebral ischemia injury likely involve the CaMKKβ/AMPK/mTOR pathway.

### ST2-104 peptide protected against Glu-induced SH-SY5Y cell death

**Fig. 4:**
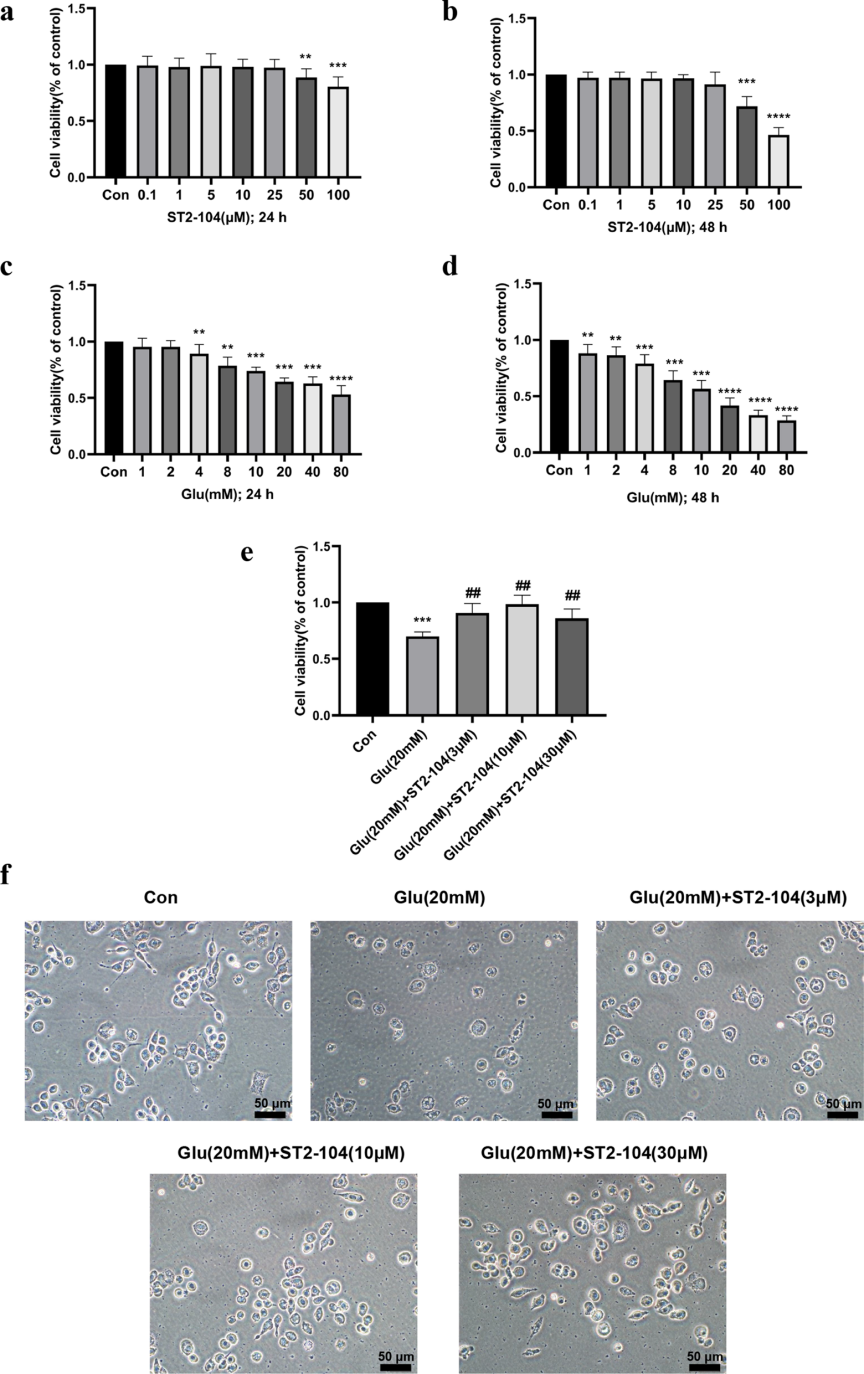
Prevention of excitotoxic death with ST2-104. SH-SHY5Y cells were treated with 20 mM glutamate plus 20 μM glycine or control medium or with ST2-104 peptide for 24 or 48 h at 37 °C and then cell viability was evaluated with the MTT assay. Cell viability was measured with the indicated concentrations of ST2-104 peptide for 24 h (**a**) and 48 h (**b**). (**c**), (**d**) Cell viability was measured with indicated concentration of Glu treatment for 24 h (**c**) and 48 h (**d**). **e** Cell viability was measured with indicated concentration of ST2-104 peptide in Glu-triggered cells. Average death in each coverslip was counted in three fields. The percentage cell viability of 6 wells is represented as S.E. (*error bars*) (*n* = 6 for each condition). **f** The representative images of the cells after insult and treatment groups; scale bar, 50 μm.^**^P < 0.01, ^***^P < 0.001, ^****^P < 0.0001 vs. Con group; ^##^*P* < 0.01 vs. Glu group with one-way ANOVA with Tukey’s post-hoc test

To test if ST2-104 peptide is neuroprotective in models of glutamate-induced toxicity. SH-SY5Y cells were pretreated for 30 min with either vehicle (DMSO) or ST2-104 peptide and then stimulated for 1 h with 20 mM glutamate + 20 μM glycine, and cell viability was quantified 24 h or 48 h later with the MTT assay. Pretreatment with ST2-104 peptide for 24 h (Fig. 4a) or 48 h (Fig. 4b), at concentrations below 25 μM alone, did not affect cell viability as compared to control (DMSO-treated) cells. At 50 and 100 µM, ST2-104 peptide reduced cell viability at 24 h and more dramatically: ~ 50% viability observed in cells treated for 48 h with 100 µM ST2-104 (Fig. 4a, b). Glutamate + glycine treatment reduced cell viability in a concentration- and time-dependent manner (Fig. 4c, d). We observed a ~ 35% (at 24 h) and ~ 55% loss (at 48 h) in cell viability at a concentration of 20 mM glutamate. Cells challenged with 20 mM glutamate also became shrunken and fragmented. (Fig. 4c, d, f). Subsequently, we used the 20 mM concentration of glutamate to determine the neuroprotective effects of ST2-104. Treatment with ST2-104 peptide (3–30 μM) for 24 h remarkably protected against glutamate-induced cell death and ameliorated cell morphology, with complete protection afforded with a 10 µM concentration of ST2-104 (Fig. 4e, f). Therefore, 10 μM of ST2-104 peptide was selected for subsequent experiments.

### ST2-104 peptide ameliorates glutamate-triggered apoptotic death in SH-SY5Y cells

**Fig. 5:**
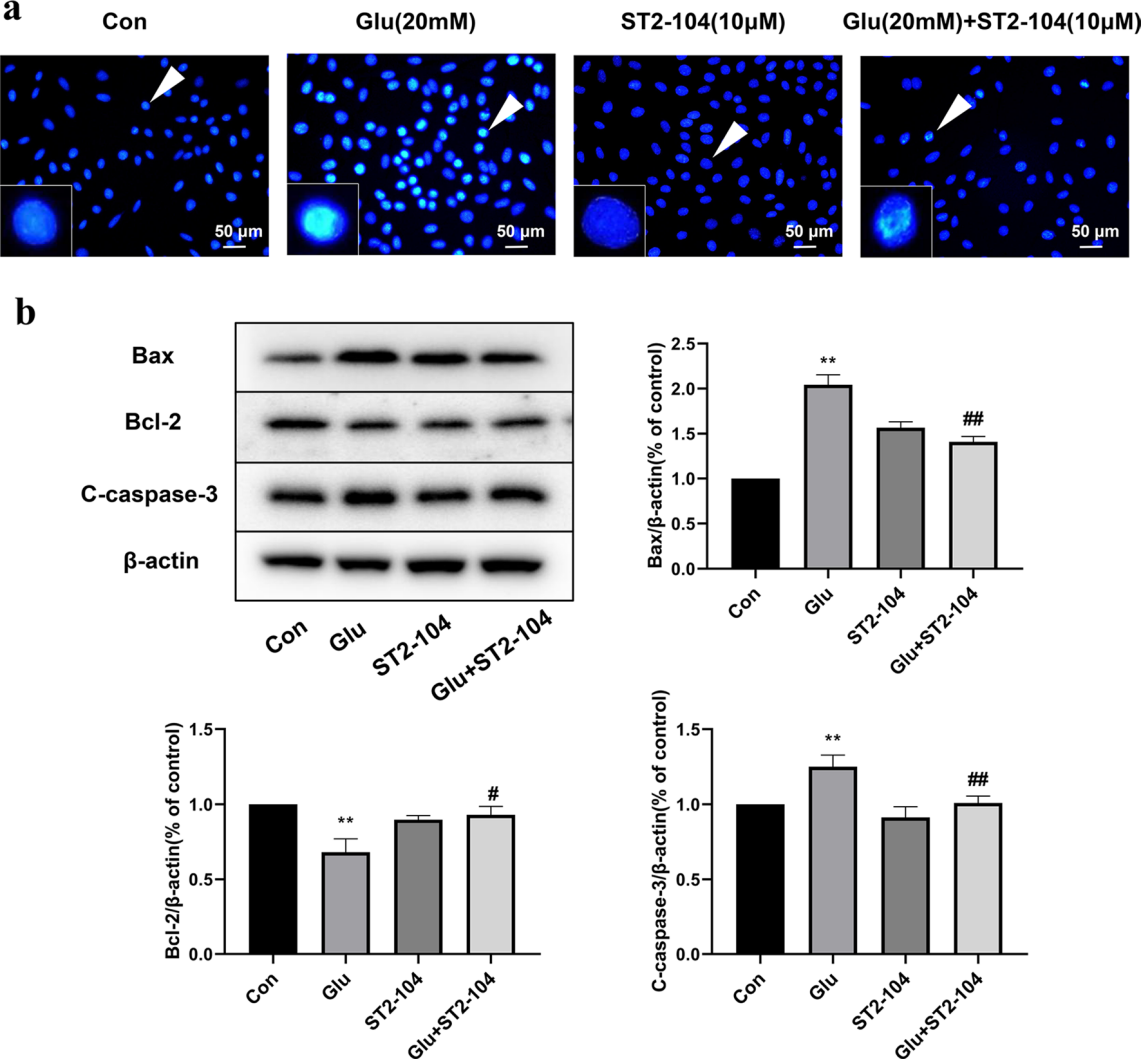
ST2-104 peptide ameliorates glutamate-induced apoptosis in SH-SY5Y cells. SH-SHY5Y cells were treated with 20 mM glutamate plus 20 μM glycine or control medium or with ST2-104 peptide for 24 h at 37 °C and then apoptosis levels and apoptotic-related proteins were assessed. **a** Representative images of apoptotic nuclei. Arrowheads indicate nuclear condensation and other morphological changes related to apoptosis; scale bar, 50 μm. For each well, at least 5 different fields were examined—a representative is shown here. **b** Detection of Bax, Bcl-2 and C-caspase-3 protein levels (representative blot is shown) using Western blot analysis. Levels of β-actin protein were used as the loading control. Bar represents mean ± SEM from 3 separate wells. ^**^P < 0.01 vs. Con group; ^#^*P* < 0.05, ^##^*P* < 0.01 vs. Glu group with one-way ANOVA with Tukey’s post-hoc test

We used Hoechst 33258 staining to evaluate whether ST2-104 peptide inhibits apoptotic cell death in SH-SY5Y cell death. Treatment of cells with 20 mM glutamate induced nuclear condensation and other morphological changes related to apoptosis compared with to control group (Fig. 5a). ST2-104 peptide (10 μM) normalized cell morphology and reduced the apoptotic activity in comparison to the glutamate-treated group (Fig. 5a).

Next, we evaluated changes of apoptosis-related proteins Bax, C-caspase-3 and Bcl-2 by Western blot. Compared to the control group, glutamate challenge increased the expression of Bax and C-caspase-3 and decreased expression of Bcl-2 (Fig. 5b). The ST2-104 peptide blocked these glutamate-induced changes while having no effect alone (i.e., in the absence of the glutamate challenge) (Fig. 5b). These results indicate that ST2-104 peptide significantly reduces excitotoxicity mediated apoptotic death in SH-SY5Y cells.

### ST2-104 peptide ameliorated glutamate-induced autophagy in SH-SY5Y cells

**Fig. 6:**
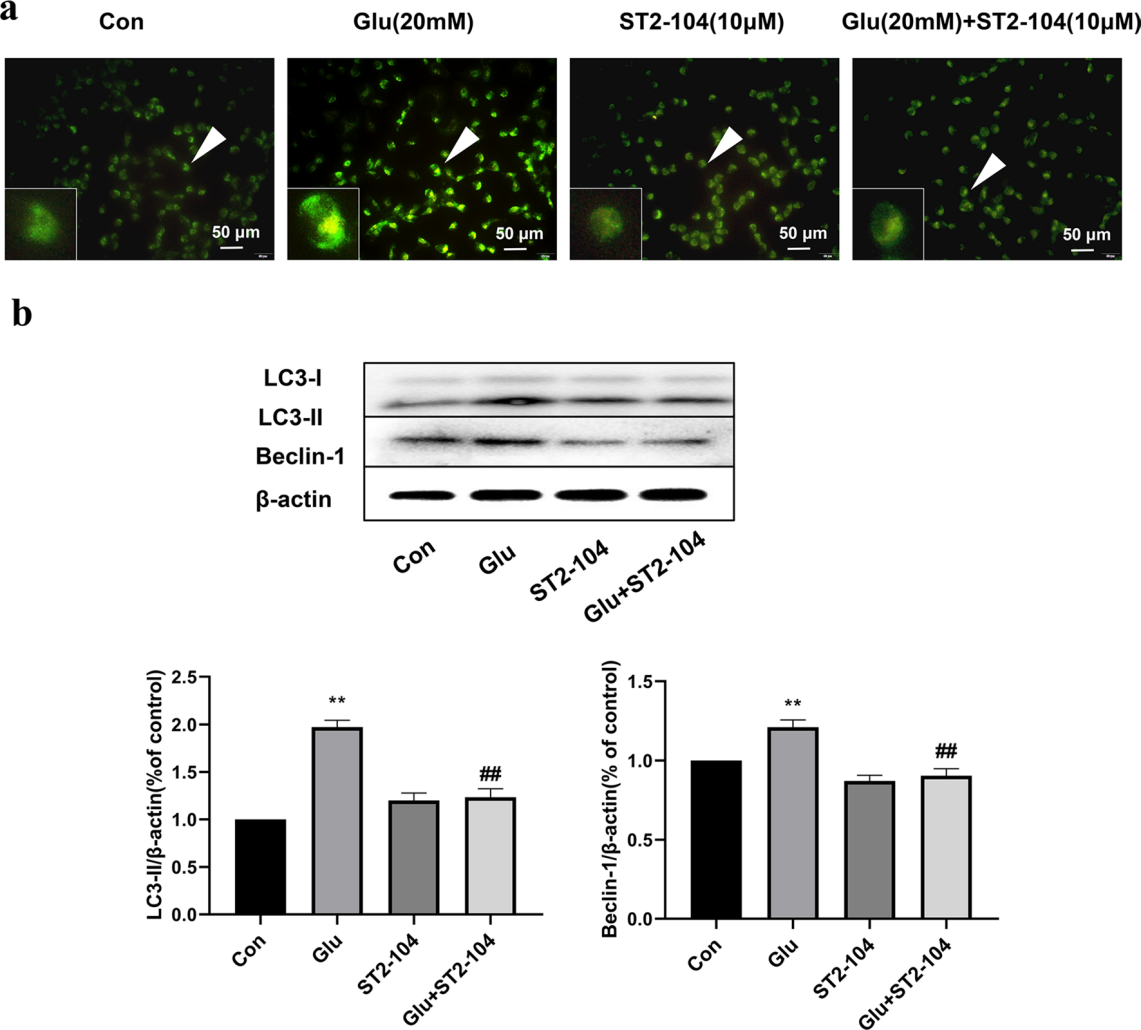
ST2-104 peptide ameliorated Glu-induced autophagy in SH-SY5Y cells. SH-SHY5Y cells were treated with 20 mM glutamate plus 20 μM glycine or control medium or with ST2-104 peptide for 24 h at 37 °C and then autophagy levels and autophagy-related proteins were assessed. **a** Representative images of autophagosomes. Arrowheads indicate autophagosomes marked by MDC staining; scale bar, 50 μm. For each well, at least 5 different fields were examined – a representative is shown here. **b** Detection of LC3-II and Beclin-1 protein levels (representative blot is shown) using Western blot analysis. Levels of β-actin protein were used as the loading control. Bar represents mean ± SEM from 3 separate wells. ^**^P < 0.01 vs. Con group; ^##^*P* < 0.01 vs. Glu group with one-way ANOVA with Tukey’s post-hoc test

Monodansylcadaverine (MDC), a specific in vivo marker for autophagic vacuoles, was used to assess autophagy in SH-SHY5Ycells. After staining with MDC under a fluorescence microscope, the autophagic vacuoles displayed green spots mainly distributed in the perineuclei. Compared to the control group, an increasing trend of autophagosomes (green signals in MDC staining) were noted in the glutamate-treated group; ST2-104 peptide decreased the number of autophagosomes (Fig. 6a). Next, we used immunoblotting to analyze the level of the autophagy-related proteins LC3-II and Beclin-1. As noted earlier, LC3-II is the membrane-bound form of the microtubule-associated protein 1A light chain 3 and serves as a proxy for the extent of autophagosome formation. Beclin 1 is an essential mediator of autophagy. Treatment with glutamate increased the amount of LC3-II and Beclin-1 in comparison to the control group, ST2-104 normalized the levels of these proteins to that observed in control of peptide-alone treated cells (Fig. 6b). These data demonstrate that ST2-104 peptide can significantly reduce glutamate-induced autophagy of SH-SY5Y cells.

### ST2-104 peptide decreases intracellular Ca^2+^ concentration induced by glutamate

**Fig. 7:**
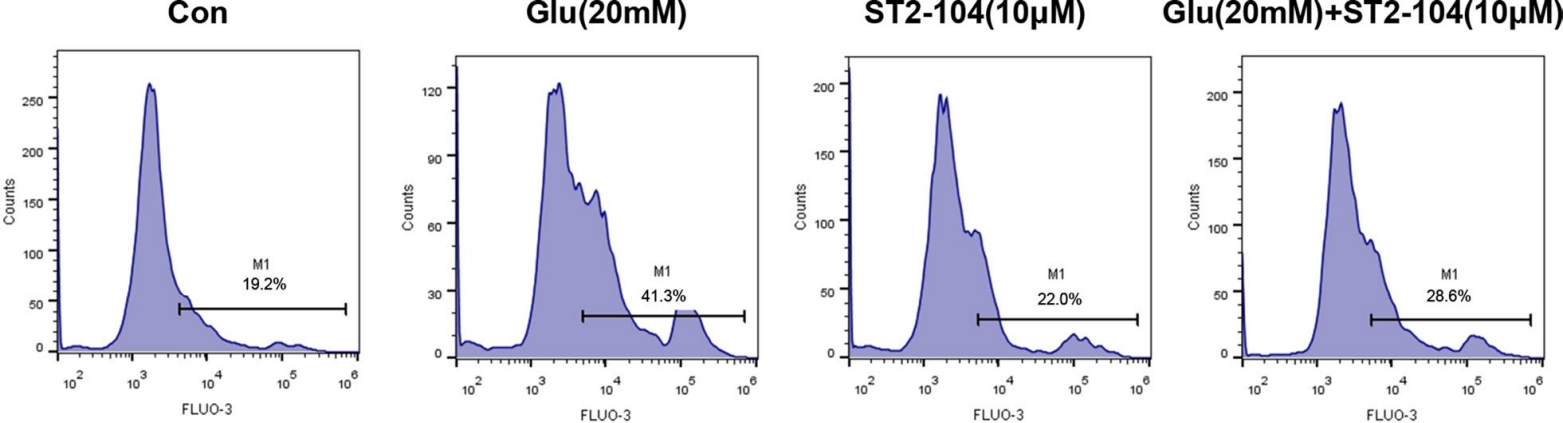
ST2-104 peptide decreases the enhancement in intracellular Ca^2+^ induced by glutamate. Intracellular calcium ([Ca^2+^]i) in SH-SY5Y cells measured by flow cytometry. SH-SHY5Y cells were treated with 20 mM glutamate plus 20 μM glycine or control medium or with ST2-104 peptide for 24 h at 37 °C and then flow cytometry was performed. [Ca^2+^]i was measured by loading the cells with 4 μM of Fluo-3/AM and examining their fluorescence intensity. The results are presented as the mean ± SEM from three independent experiments

Increases in intracellular Ca^2+^ levels have been reported in many experimental models of apoptosis. Therefore, we used the Ca^2+^-sensitive fluorescence probe Fluo-3/AM to monitor alterations in the intracellular Ca^2+^ by flow cytometry. When SH-SY5Y cells were exposed to 20 μM glutamate for 24 h, the histogram of Fluo-3 fluorescence shifted to a higher intensity (Fig. 7), indicating an increase in [Ca^2+^]_i_. In other words, the mean fluorescence intensity (MFI) and percentage of cells in gate (M1) were changed with glutamate-treatment increasing the concentration of intracellular Ca^2+^ (MFI:19,962, M1:41.3%) and while ST2-104 peptide (MFI:14,871, M1:28.6%) restoring the glutamate-enhanced Ca^2+^ levels to that observed unter control conditions (MFI:11,456, M1:19.2%) (Fig. 7). These results suggest that ST2-104 can normalize elevated [Ca^2+^]_i_.

### Reversing ST2-104 peptide-mediated inhibition of autophagy aggravated apoptotic cell death in SH-SY5Y cells

To further explore the relationship between autophagy and apoptosis in ST2-104 peptide-mediated neuroprotection effects, we used ST2-104 peptide combined with rapamycin (RAPA) to treat SH-SY5Y cells. Rapamycin is a macrolide immunosuppressant that inhibits the mechanistic target of rapamycin (mTOR) protein kinase. Apoptotic levels were evaluated by Hoechst 33258 fluorescent staining.

**Fig. 8:**
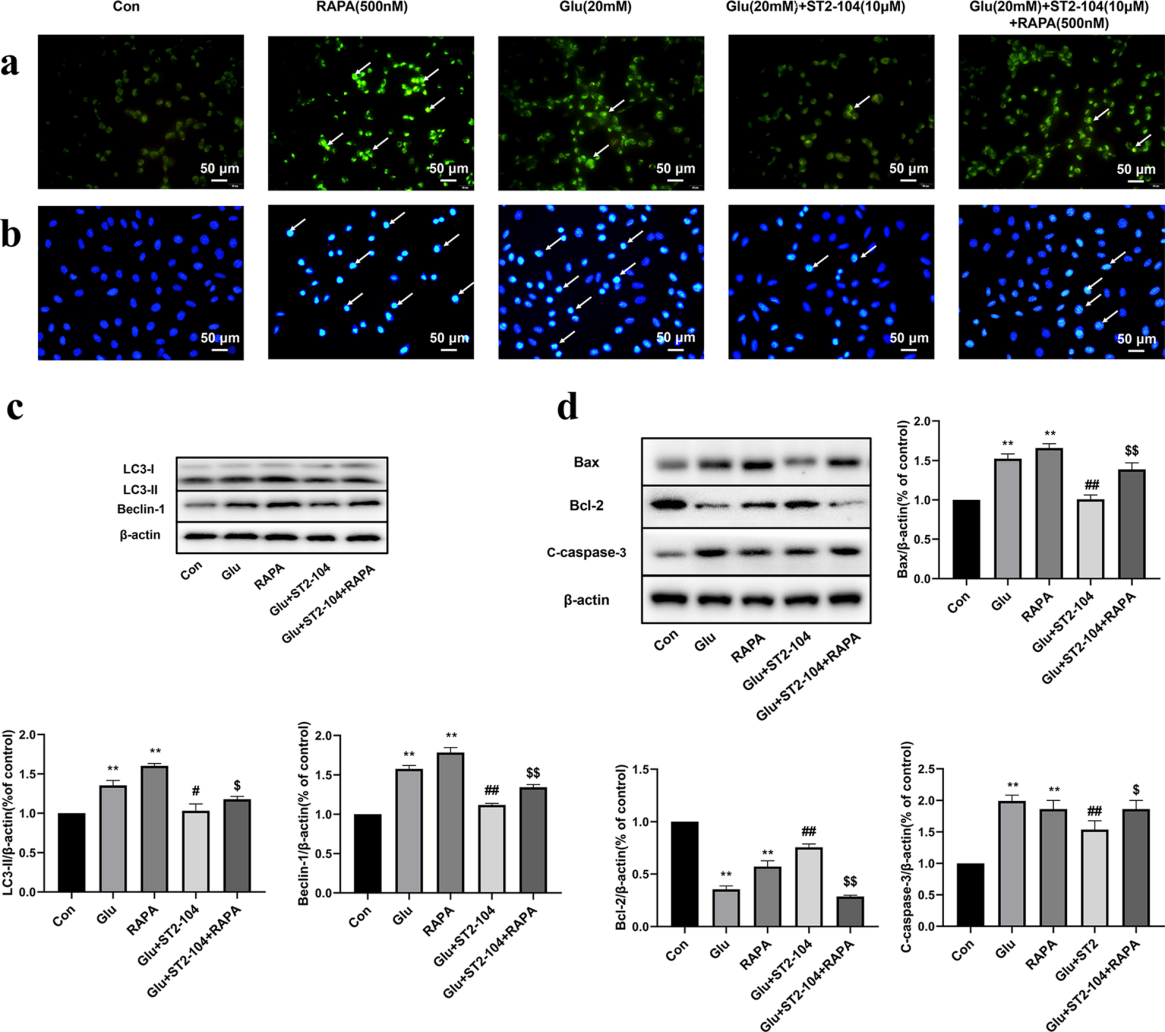
Reversing ST2-104 peptide-mediated inhibition of autophagy exacerbates apoptotic cell death in SH-SY5Y cells. SH-SHY5Y cells were treated with 20 mM glutamate plus 20 mM glycine or control medium or with ST2-104 peptide for 24 h at 37 °C and then apoptosis and autophagy levels and apoptosis- and autophagy-related proteins were assessed. **a** Representative images of autophagosomes. Arrows indicate autophagosomes marked by MDC staining; scale bar, 50 μm. For each well, at least 5 different fields were examined – a representative is shown here. **b** Apoptosis level was evaluated using the Hoechst 33,258 staining. Scale bar: 50 μm. **c**, **d** Detection of LC3-II, Beclin-1, Bax, Bcl-2 and C-caspase-3 protein expression levels using Western blot analysis. Representative blots are shown. Levels of β-actin protein were used as the loading control. Bar represents mean ± SEM from 3 separate wells. ^**^P < 0.01, vs. Con group; ^#^*P* < 0.05, ^##^*P* < 0.01, vs. Glu group; ^$^*P* < 0.05, ^$$^*P* < 0.01, vs. Glu + ST2-104 peptide group with one-way ANOVA with Tukey’s post-hoc test

Consistent with earlier data, autophagy triggered by glutamate challenge was reduced by ST2-104 but RAPA reversed this effect (Fig. 8a). Additionally, as evaluated by Hoechst 33258 staining, ST2-104 attenuated the level of apoptosis whereas ST2-104 co-applied with RAPA reversed the reduction (Fig. 8b). Next, we used immunoblots to analyze the expression of the autophagy-related proteins LC3-II and Beclin-1 and apoptosis-related proteins Bax, C-caspase-3 and Bcl-2. SH-SHY5Y cells challenged with 20 mM glutamate plus 20 μM glycine had elevated levels of LC3-II and Beclin-1 which were reduced by ST2-104 but RAPA negated this effect; RAPA alone also increased LC3-II and Beclin-1 (Fig. 8c). Glutamate treated SH-SHY5Y cells had increased levels of Bax and C-caspase-3 and decreased levels of Bcl-2, these effects were reversed by ST2-104, but cancelled by RAPA co-treatment (Fig. 8d). Thus, these data show that the autophagy activator RAPA can block the protective effect of ST2-104 peptide on glutamate-induced apoptosis, suggesting that ST2-104 peptide can block glutamate-induced apoptosis by inhibiting autophagy.

### Enhancing ST2-104 peptide-mediated inhibition of autophagy ameliorates apoptotic cell death in SH-SY5Y cells

**Fig. 9:**
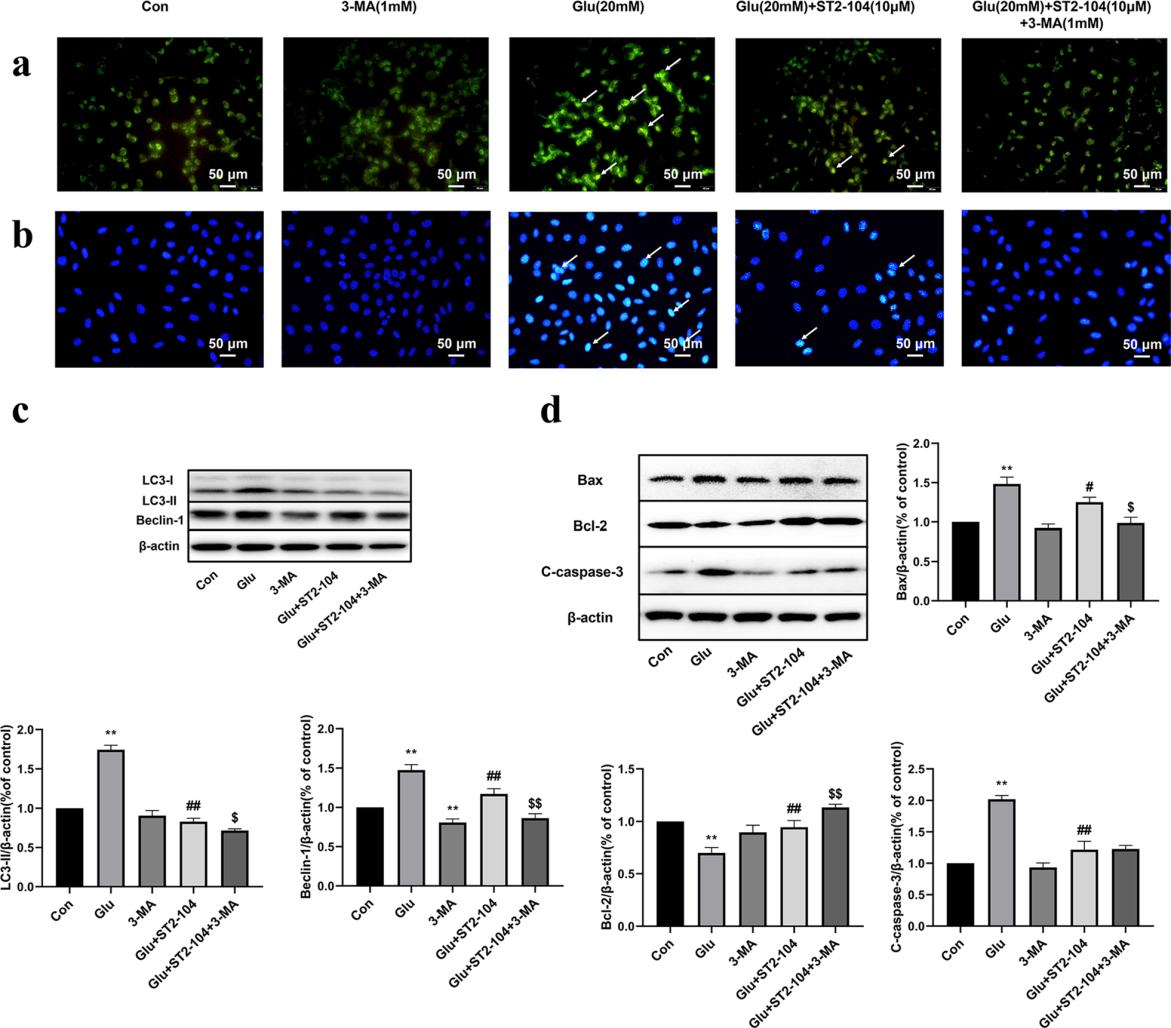
Enhancing ST2-104 peptide-mediated inhibition of autophagy ameliorates apoptotic cell death in SH-SY5Y cells. SH-SHY5Y cells were treated with 20 mM glutamate plus 20 μM glycine or control medium or with ST2-104 peptide for 24 h at 37 °C and then apoptosis and autophagy levels and apoptosis- and autophagy-related proteins were assessed. **a** Representative images of autophagosomes. Arrows indicate autophagosomes marked by MDC staining; scale bar, 50 μm. For each well, at least 5 different fields were examined – a representative is shown here. **b** Apoptosis level was evaluated using the Hoechst 33,258 staining. Scale bar: 50 μm. **c**, **d** Detection of LC3-II, Beclin-1, Bax, Bcl-2 and C-caspase-3 protein expression levels using Western blot analysis. Representative blots are shown. Levels of β-actin protein were used as the loading control. Bar represents mean ± SEM from 3 separate wells. ^**^P < 0.01, vs. Con group; ^#^*P* < 0.05, ^##^*P* < 0.01, vs. Glu group; ^$^*P* < 0.05, ^$$^*P* < 0.01, vs. Glu + ST2-104 peptide group with one-way ANOVA with Tukey’s post-hoc test

To interrogate possible crosstalk between autophagy and apoptosis in ST2-104 peptide-mediated neuroprotection, SH-SY5Y cells were pretreated with ST2-104 peptide in combination with 3-Methyladenine (3-MA), an autophagy inhibitor and then the apoptosis and autophagy levels were assessed as before. As observed with MDC staining, combining ST2-104 peptide with 3-MA reduced autophagy levels in the face of a glutamate-challenge (Fig. 9a). Similarly, as expected, the number of apoptotic SH-SY5Y cells found in the glutamate-treated condition were decreased by ST2-104 peptide and co-adding 3-MA to the cells further suppressed apoptosis (Fig. 9b). Commensurate with this result, the autophagy-related proteins LC3-II and Beclin-1 were decreased by ST2-104 peptide alone or in combination with 3-MA (Fig. 9c). Glutamate treated SH-SHY5Y cells had increased levels of Bax and C-caspase-3 and decreased levels of Bcl-2, these effects were again reversed by ST2-104 peptide alone or in combination with 3-MA (Fig. 9d). Collectively, these data demonstrate that the autophagy inhibitor 3-MA can promote the protective role of ST2-104 peptide against glutamate-induced apoptosis, reaffirming that ST2-104 dampens glutamate-induced apoptosis by inhibiting autophagy.

### CaMKKβ levels are regulated by ST2-104 to reduce apoptosis

**Fig. 10:**
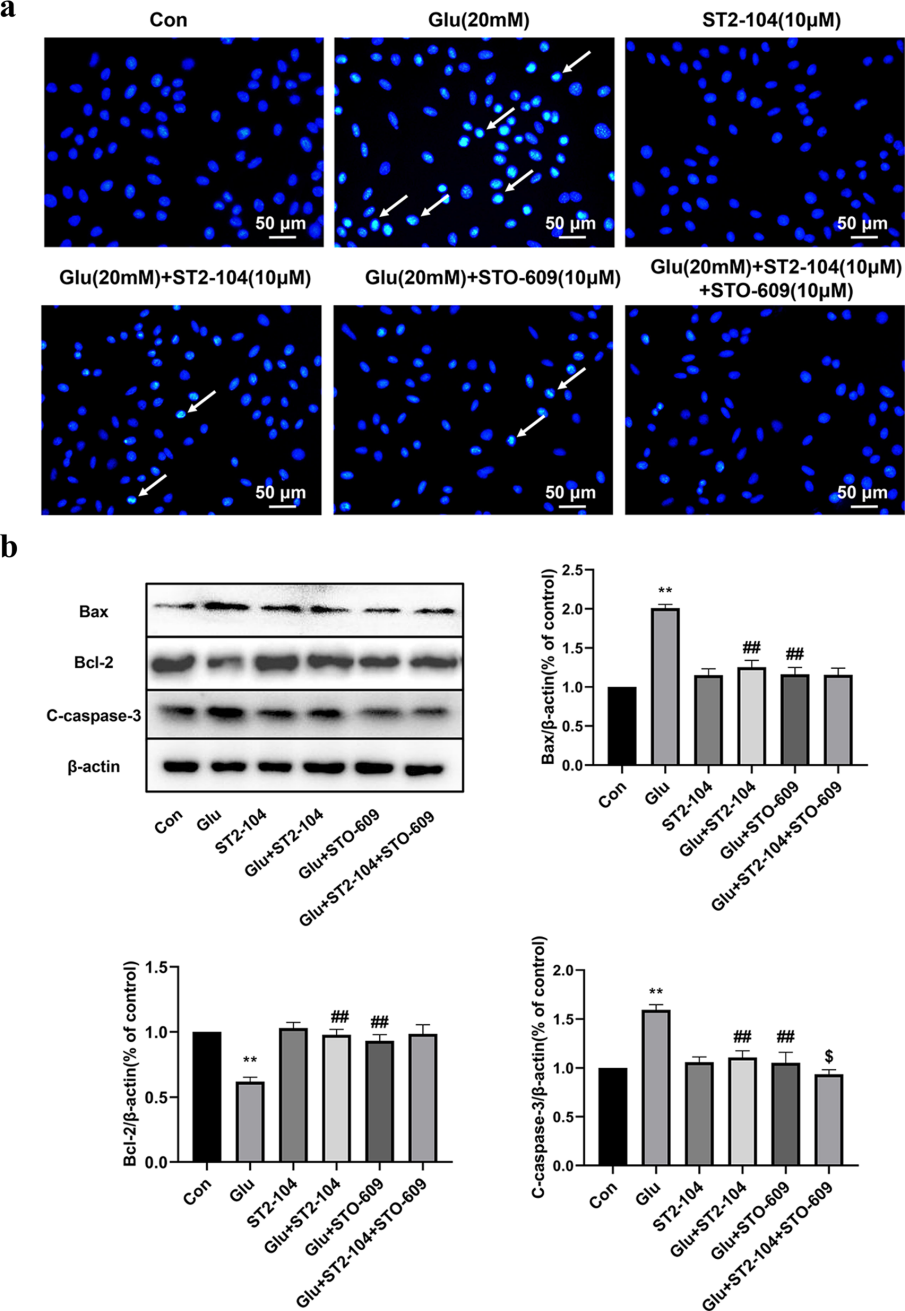
Role of CaMKKβ in the ST2-104-mediated decline in apoptosis. SH-SHY5Y cells were treated with 20 mM glutamate plus 20 μM glycine or control medium or with ST2-104 peptide for 24 h at 37 °C and then apoptosis levels and apoptosis-related proteins were assessed. In some wells, 10 μM STO-609, an inhibitor of CaMKKβ was added for 24 h. **a** Apoptosis level was evaluated using the Hoechst 33,258 staining. Scale bar: 50 μm. For each well, at least 5 different fields were examined – a representative is shown here. **b** Detection of Bax, Bcl-2 and C-caspase-3 protein expression levels using Western blot analysis. Representative blots are shown. Levels of β-actin protein were used as the loading control. Bar represents mean ± SEM from 3 separate wells. ^**^P < 0.01, vs. Con group; ^##^*P* < 0.01, vs. Glu group; ^$^*P* < 0.05, vs. Glu + ST2-104 peptide group with one-way ANOVA with Tukey’s post-hoc test

It has been reported that a Ca^2+^/CaMKKβ/AMPK/mTOR signaling platform contributes to regulation and inhibition of autophagy.To determine the involvement of CaMKKβ in ST2-104 peptide-mediated apoptosis, the CaMKKβ inhibitor ST0-609 was utilized. ST2-104 decreased glutamate-induced apoptosis and this protective effect was enhanced by STO-609 (Fig. 10a). At the protein level, STO-609 decreased the expression of Bax and C-caspase-3 and increased the expression of Bcl-2 significantly compared with glutamate-treated group, similar to changes brought about by ST2-104 (Fig. 10b). Therefore, ST2-104 peptide inhibits the apoptosis of SH-SY5Y cells by regulating CaMKKβ.

### Role of CaMKKβ in the ST2-104-mediated decline in autophagy

**Fig. 11:**
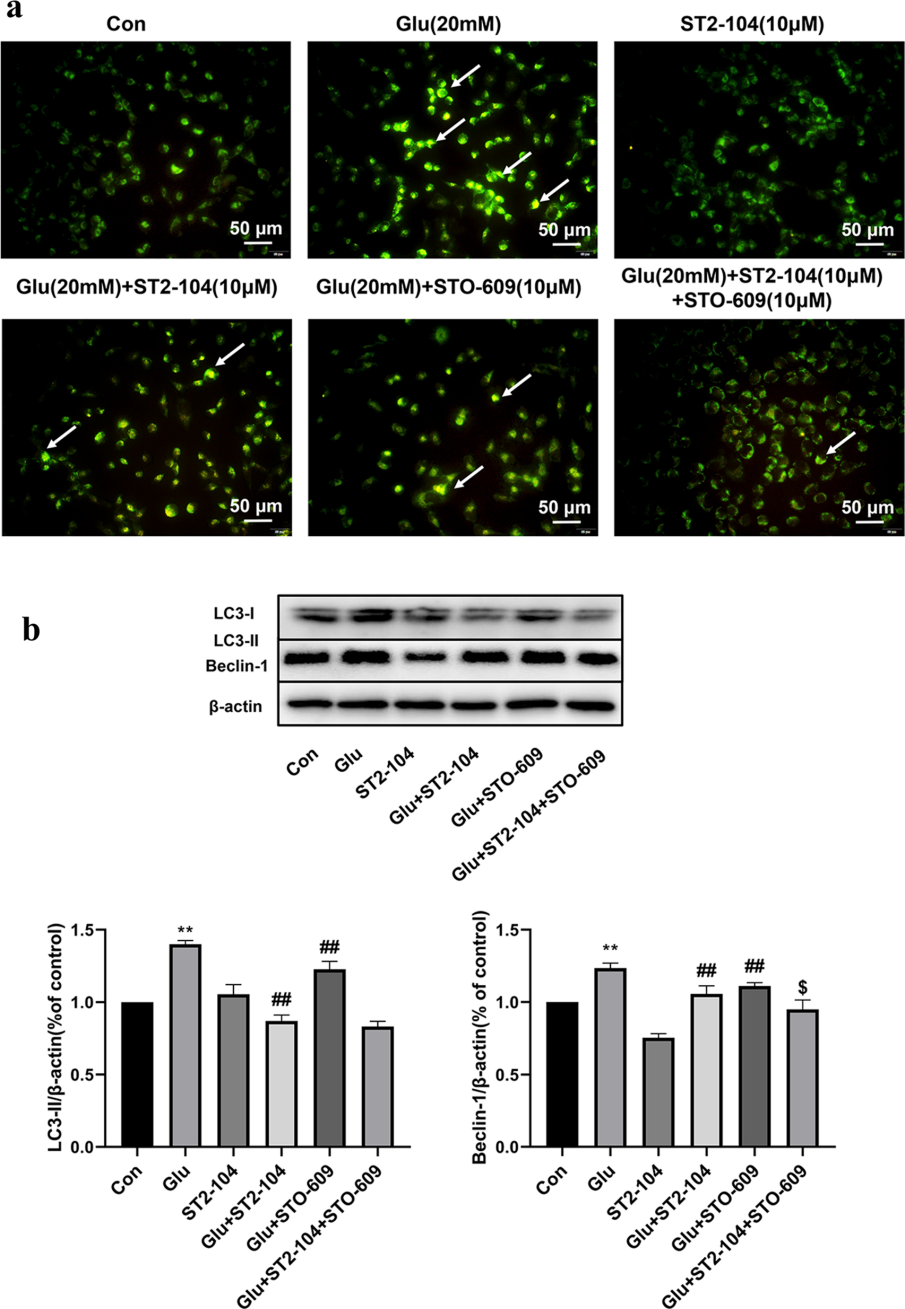
Role of CaMKKβ in the ST2-104-mediated decline in autophagy. SH-SHY5Y cells were treated with 20 mM glutamate plus 20 μM glycine or control medium or with ST2-104 peptide for 24 h at 37 °C and then autophagy levels and autophagy-related proteins were assessed. In some wells, 10 μM STO-609, an inhibitor of CaMKKβ was added for 24 h. **a** Autophagy level was evaluated using MDC staining. Scale bar: 50 μm. For each well, at least 5 different fields were examined – a representative is shown here. **b** Detection of LC3 and Beclin -1 protein expression levels using Western blot analysis. Representative blots are shown. Levels of β-actin protein were used as the loading control. Bar represents mean ± SEM from 3 separate wells. ^**^P < 0.01, vs. Con group; ^##^*P* < 0.01, vs. Glu group; ^$^*P* < 0.05, vs. Glu + ST2-104 peptide group with one-way ANOVA with Tukey’s post-hoc test

Next, we asked if CaMKKβ could also contribute to glutamate-triggered autophagy in SH-SY5Y cells. As earlier, to determine the involvement of CaMKKβ in ST2-104 peptide-mediated autophagy, the CaMKKβ inhibitor ST0-609 was utilized. ST2-104 decreased glutamate-induced autophagy and this protective effect was enhanced by STO-609 (Fig. 11a). At the protein level, STO-609 decreased the expression of LC3-II and Beclin-1 level (Fig. 11b). Therefore, it appears that ST2-104 peptide inhibits the autophagy of SH-SY5Y cells by blocking CaMKKβ.

### ST2-104 peptide regulates a CaMKKβ/AMPK/mTOR pathway in glutamate-challenged SH-SY5Y cells

**Fig. 12:**
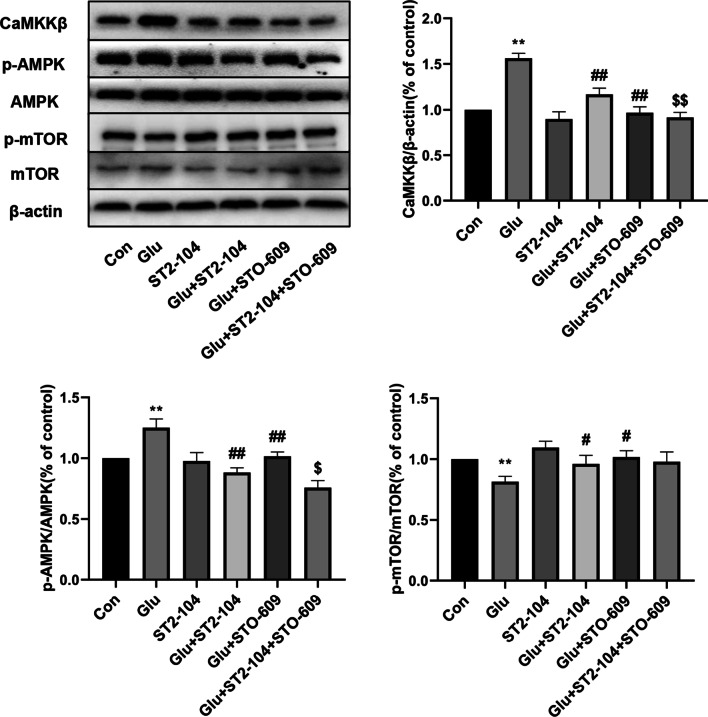
Involvement of a CaMKKβ/AMPK/mTOR pathway in the effects of ST2-104. SH-SHY5Y cells were treated with 20 mM glutamate plus 20 μM glycine or control medium or with ST2-104 peptide for 24 h at 37 °C and then protein levels were assessed by immunoblotting. Detection of CaMKKβ, AMPK, pAMPK, mTOR and p-mTOR protein expression levels using Western blot analysis. Representative blots are shown. Levels of β-actin protein were used as the loading control. Bar represents mean ± SEM from 3 separate wells. ^**^P < 0.01, vs. Con group; ^#^*P* < 0.05, ^##^*P* < 0.01, vs. Glu group; ^$^*P* < 0.05, ^$$^*P* < 0.01 vs. Glu + ST2-104 peptide group with one-way ANOVA with Tukey’s post-hoc test

Finally, we explored the molecular mechanisms of ST2-104 peptide in glutamate-challenged SH-SY5Y cells. To test if CaMKKβ and downstream signaling molecules participated in curbing autophagy, SH-SY5Y cells were treated with the CaMKKβ inhibitor STO-609 (10 µM) and the protein levels of pathway related proteins CaMKKβ, p-AMPK, and p-mTOR were assessed by Western blot. ST2-104 peptide reduced Glu-induced autophagy by down regulating LC3-II and Beclin-1 level, CaMKKβ level and p-AMPK/AMPK ratio, while up regulating p-mTOR/mTOR ratio. STO-609 further enhanced these effects of ST2-104 peptide in protein expression (Fig. 12). Thus, these data support a role for ST2-104 peptide in attenuating a CaMKKβ/AMPK/mTOR pathway in SH-SY5Y cells.

## Discussion

Cerebral ischemia, along with heart disease and cancer, are the leading causes of death and physical disability worldwide [35]. Thrombolytic therapy such as recombinant tissue plasminogen activator has been the standard of care for treatment of acute ischemic stroke for several years [36]. However, due to its contraindications such as bleeding, effects on blood pressure, seizure and other risks, new therapeutic strategies must be considered. Our work has identified a short peptide (ST2-104 or R9-CBD3) from the axon guidance and outgrowth protein collapsin response mediator protein 2 (CRMP2) that has actions on calcium channels (CaV2.2) [26, 37], sodium-calcium exchangers (NCX3) [24], and receptors (e.g. N-methyl-D-aspartate receptors (NMDARs) [23, 24, 38]. We also reported that ST2-104 peptide can reduce calcium overload, inhibit neuronal apoptosis and reduce amyloid beta (Aβ)-induced spatial cognitive and memory impairment in AD rats, and showed neuroprotective effects on other CNS diseases [23]. Inspired by these promising neuroprotective effects of ST2-104, here we further explored the effects of this peptide on cerebral ischemia injury in MCAO model in vivo and glutamate-induced SH-SY5Y cells injury in vitro. Our results demonstrate that ST2-104 peptide protects against sensorimotor function loss as well neuronal death via inhibition of apoptosis and autophagy. While we did not interrogate if ST2-104 has any effects on immune cells, CBD3 when administered in a viral vector to animals did not affect inflammatory responses [39].

Cumulative evidence suggests that apoptotic cell death occurs in cerebral ischemia models [40]. Sun and colleagues reported that TUNEL positive cells increased 24 h following cerebral ischemia. Ischemia/reperfusion induced neuronal apoptosis as inferred from increased expression levels of Bax and cytochrome-c in the cerebral cortex [41]. Activation of caspase-3 can be enhanced by its initiators such as caspase-11; in caspase-11 knockout animals, the level of apoptosis is decreased following focal ischemia [42]. In this study, we found that MCAO and glutamate induced apoptosis in vivo and in vitro, respectively. Importantly, ST2-104 peptide pretreatment decreased expression of Bax and C-caspase-3 but increased Bcl-2 expression and reduced fluorescence intensity in Hoechst 33258 staining (a proxy for apoptosis), demonstrating that the neuroprotective actions of ST2-104 peptide rely on blocking neuronal apoptosis.

Autophagy is widely acknowledged as a mechanism for maintaining neuronal homeostasis in the central nervous system [43, 44]. When homeostasis is impaired, organelles increase, dysfunctional proteins accumulate, or outside pathogens invade, and as a result vesicular membrane structures start to stretch, wrap cell contents and integrate with lysosomes to form autolysosomes [45]. Subsequently, the cell contents are degraded to amino acid and other small molecules that can be recycled by the cell. Mild autophagy activation is a neuroprotective response, but cerebral ischemic damage caused by excessive autophagy has also been reported. The study by Huang and co-workers demonstrated that crocin (the chemical found in saffron flowers) induces anti-ischemia in MCAO rats and inhibits autophagy by regulating mTOR [46]. TIGAR, an enzyme that functions mainly as a regulator of glucose breakdown in human cells, relieves neuronal death by restraining autophagy and rapamycin partially abolishes this neuroprotection. In addition, a mTOR-S6KP70 signaling pathway has been implicated in this process [47]. These studies have shown that autophagy has negative effects on cerebral ischemia/reperfusion injury. However, autophagy also reported to be neuroprotective in ischemic brain [48] which might be due to different ischemic models and various animal strains. Therefore, it is still a controversial issue whether autophagy contributes to cell survival. This study showed that autophagy exacerbated brain ischemia in rats and that the ST2-104 peptide-mediated suppression of autophagy could further reduce infarct size and improve neurological function. Similarly, the glutamate-induced increased in autophagosomes in SH-SY5Y cells was reduced by ST2-104 peptide. Taken together, inhibition of autophagy leads to the protective effects of ST2-104 peptide in cerebral ischemia/stroke.

Autophagy is a highly conserved lysosomal degradation pathway that is controlled by numerous proteins and genes. Beclin-1, an essential regulator of autophagy, is homologous with ATG6 in yeast which facilitates the generation of autophagosome membranes during the initial stage of autophagy [49]. LC3, another biological marker of autophagy during autophagosome formation, has two subtypes. LC3-I (cytoplasmic type) is transformed into LC3-II (membrane type) by ATG4-mediated dihydroxylation when autophagosomes are forming [50]. Per our findings, ST2-104 peptide pretreatment further reversed MCAO or glutamate induced enhancement of Beclin-1 and LC3II expression, suggesting that the protective function of ST2-104 peptide has a close relationship with autophagy inhibition in cerebral ischemia injury.

It has been reported that apoptosis is closely linked to autophagy [51, 52]. Previous studies have shown that apoptosis processes could be boosted by excessive autophagy during transient global cerebral ischemia. Moreover, studies also proved that knocking out the ATG7 gene could effectively decrease the activation of caspase-3 and inhibit cell apoptosis in neonatal mice with cerebral ischemia injury [53]. It was also found in MCAO rats that the level of Bcl-2 increases following treatment with 3-MA, a molecule that inhibits autophagy [41]. Based on these studies, a potential correlation between autophagy and apoptosis was further explored here. We used rapamycin (autophagy enhancer) and 3-MA (autophagic inhibitor) to interrogate this link in treat SH-SY5Y cells. The results revealed that 3-MA promotes the inhibitory effects of ST2-104 peptide against apoptosis while RAPA counteracts the aforementioned effects in glutamate-treated cells. Therefore, ST2-104 peptide may protect against apoptosis induced by glutamate by inhibiting autophagy. Since glutamate has been reported to inhibit cystine/glutamate antiporter system xc^−^ in SH-SY5Y neuroblastoma cells via to induce oxytosis or ferroptosis [54], we cannot exclude the possibility that ST2-104 may work thorough this pathway.

Here we investigated the molecular mechanisms of ST2-104 peptide in regulating autophagy. Multiple signaling pathways contribute to the regulation of autophagy and CaMKKβ/AMPK/mTOR is one of them [19]. Ca^2+^ has been demonstrated to be closely correlated with autophagy. Current studies have shown that CaMKKβ could be activated by accumulation of intracellular Ca^2+^ concentration, leading to downstream AMPK signaling activation. AMPK, an energy sensor and a metabolism regulator, is sensitive to the change of AMP/ATP ratio. Under ischemia and hypoxia, AMPK promotes autophagy directly by phosphorylating ULK1. Suppression of autophagy mediated by reduced activation of AMPK is favorable in ischemia stroke. mTOR is a key downstream target of AMPK that affects protein translation, ribosome synthesis and other metabolic processes. Activation of mTORC1-4E-BP or inhibition of TSC2-mTOR-S6K1 participates in the autophagic damage of neurons [55, 56]. In this study, the level of CaMKKβ and p-AMPK were increased, while p-mTOR was reduced by MCAO or glutamate; these changes were blunted by ST2-104 peptide. The increased intracellular Ca^2+^ concentration in response to treatment with glutamate was also reduced by ST2-104 peptide in SH-SY5Y cells. Additionally, ST2-104 peptide reversed Glu-induced apoptosis via inhibiting CaMKKβ-mediated autophagy, which was partly enhanced by STO-609 (an inhibitor of CaMKKβ).

## Conclusion

In summary, our results suggest that the neuroprotective mechanisms of ST2-104 peptide related to preventing apoptotic death following cerebral ischemia injury can be also attributed to inhibition of autophagy via control of a CaMKKβ/AMPK/mTOR signaling pathway. In future studies, a systematic molecular manipulation of the pathways using editing techniques (e.g., CRISPR/Cas9, siRNA etc.) will enable validation of the pharmacological manipulations undertaken here. Nevertheless, the findings present novel insights into the potential neuroprotection of ST2-104 peptide in cerebral ischemia.

## Data Availability

Please contact author for data requests.

## Abbreviations

CRMP2: Collapsin response mediator protein 2
MCAO: Middle cerebral artery occlusion
CBD3: Channel-binding domain 3
CaMKKβ: Ca^2^^+^/CaM-dependent protein kinase kinase β
3-MA: 3-Methyladenine
Glu: Glutamate
Bcl-2: B-cell leukemia-2
Bax: Bcl-2-associated X protein
AMPK: AMP-activated protein kinase
mTOR: Mammalian target of rapamycin
